# Hydralazine-Associated Vasculitis and Pulmonary Hemorrhage

**DOI:** 10.7759/cureus.37332

**Published:** 2023-04-09

**Authors:** Kateryna Strubchevska, Marko Kozyk, Matthew R Jacobs, David White

**Affiliations:** 1 Internal Medicine, Beaumont Hospital, Royal Oak, USA; 2 Nephrology, Beaumont Hospital, Royal Oak, USA

**Keywords:** anca-associated vasculitis, anca, vasculitis, hypertension, pulmonary hemorrhage, hydralazine-induced vasculitis

## Abstract

Hydralazine is a potent vasodilating medication used as adjunctive therapy for the treatment of hypertension. Rarely, hydralazine may cause the development of antineutrophil cytoplasmic antibody vasculitis with the pulmonary-renal syndrome. We are presenting a case of hydralazine-associated vasculitis and pulmonary hemorrhage.

## Introduction

Hydralazine is a direct smooth muscle vasodilator used as an adjunctive medication for the treatment of hypertension, hypertensive emergencies, and heart failure with reduced ejection fraction. Rare side effect such as hydralazine-induced lupus syndrome was initially reported in 1953. The syndrome develops in 5%-10% of patients taking this medication and usually presents with arthralgias, myalgias, fever, and serositis. Rarely, renal, pulmonary, visceral, and central nervous systems involvement have been reported. Interestingly, hydralazine may trigger the development of antinuclear cytoplasmic antibody (ANCA) vasculitis, which is frequently associated with kidney involvement. Pathogenesis of the syndrome was not very well explained; however, theories include the production of antibodies to the neutrophils after binding of hydralazine with myeloperoxidase, increased expression of neutrophil autoantigens secondary to hydralazine-induced reversal of epigenetic silencing of myeloperoxidase (MPO) and proteinase 3, and problems with tolerance in slow versus fast acetylators of the medication [1].

## Case presentation

**Figure 1:**
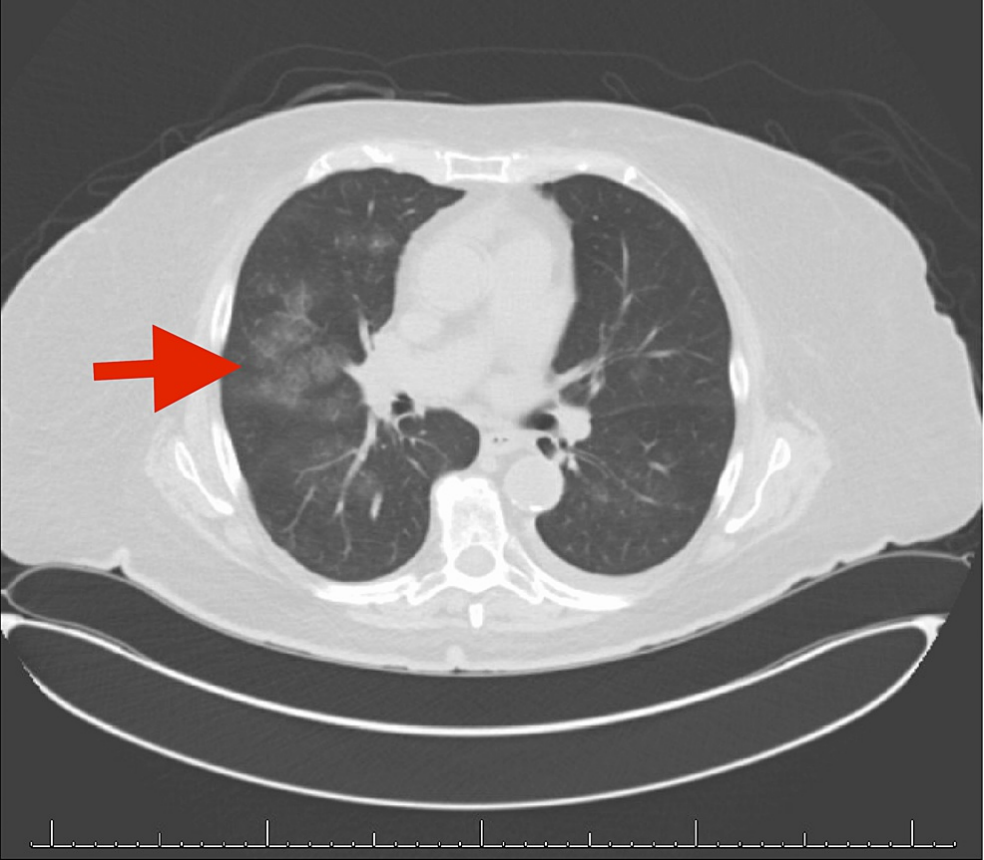
CT scan of the chest revealing pulmonary hemorrhage.

This is a 75-year-old Caucasian woman with a past medical history of hypertension, obesity, and crystal-induced septic arthritis of the right ankle treated with intravenous cefepime 2 g and vancomycin for three days who presented due to abnormal laboratory test results. The patient had an appointment with her primary care physician a few days prior to admission when she underwent her annual lab workup. She was noted to have an elevated creatinine of up to 1.35 mg/dL without a history of chronic kidney disease. A urinalysis revealed new proteinuria, hematuria, pyuria, and granular casts. Her albumin to creatinine ratio was 157 mg/g and also a new finding. Further immunologic studies revealed high positive ANA titer at 1:640, pANCA at ≥1:640, anti-double stranded antibodies at 102 AU/mL, anti-myeloperoxidase at 109 units, antiproteinase 3 at 24 units, and positive antiphospholipid antibodies (anti-cardiolipin and anti-b2 antibodies) with a drop in hemoglobin of 8.1 g/dL. Anti-histone antibodies, Sjogren SS A and B antibodies, anti-Smith antibodies, anti-RNP antibodies, and glomerular basement membrane antibodies were all negative. Noted complement C3 level was slightly decreased, and the C4 level was normal. She was admitted to the hospital for further workup to include a kidney biopsy. The patient had stable vital signs on presentation. Her review of symptoms was positive for mild pain in the left ankle with swelling, and she was concerned about another septic joint. She did not have any other arthralgias or new rash. The patient reported decreased urinary output and a noticed change in color over the last week and a half. Denied any cough, dyspnea with or without exertion, fevers, night sweats, or hemoptysis. She did admit to a 40-pound intentional weight loss over the last 6 months. Also admitted to feeling more tired and lack of energy that she attributed to stress. Of note, the patient was taking hydralazine, 100 mg BID, for blood pressure control for at least the last eight months. She did not take any other medications for her hypertension. The patient was suspected to have acute nephritic syndrome with acute kidney injury secondary to either glomerulonephritis, vasculitis, hydralazine-induced, or idiopathic. Due to the high ANCA, MPO, ds-DNA, and ANA and despite negative anti-histone antibodies, it was felt to be hydralazine induced. The patient was started on methylprednisolone 500 mg intravenous piggyback (IVPB) daily for three doses. Renal ultrasound was performed, with slight echogenicity but otherwise unremarkable. The right kidney biopsy was obtained and later revealed pauci-immune, ANCA-associated crescentic glomerulonephritis, mild acute tubular injury with mild-to-moderate interstitial fibrosis, and tubular loss. Chest CT revealed bilateral ground-glass opacities in the lungs concerning diffuse alveolar hemorrhage (DAH) or infection (Figure 1).

Bronchoalveolar lavage was performed with a small amount of blood that cleared quickly, inconsistent with DAH. Lower respiratory cultures were sent (prior to starting immunosuppressive agents) and came back negative. Urine culture was positive for *Escherichia coli* and treated successfully with ceftriaxone. The patient was then treated with one dose of intravenous cyclophosphamide as initiation therapy at 10 mg/kg due to age. It was then decided, after much collaboration with multiple nephrologists, to treat this more aggressively with dual therapy, and the patient also received 1 g of rituximab. A second dose was scheduled in two weeks to be infused outpatient but typically does not require maintenance therapy if hydralazine induced. Subsequently, she was also started on a course of tapering prednisone at 1 mg/kg and Bactrim DS thrice weekly for prophylaxis. She was also advised to receive age-appropriate vaccinations, including pneumococcus, influenza, COVID-19, and herpes zoster with an increased risk of infection. The patient acknowledged to discontinue hydralazine forever and discuss with her family the possible genetic predisposition to the drug. She was discharged home with a scheduled close outpatient follow-up in the nephrology clinic.

## Discussion

Hydralazine-induced vasculitis is an inflammation of blood vessels that results in damage of blood vessels walls. Subsequently, organs supplied by the impaired vessels experience a lack of oxygen and nutrients. The pathogenesis of the disease is not well understood due to its complexity. Probably, activated neutrophils in the presence of hydrogen peroxide release MPO from the granules and convert hydralazine into a cytotoxic product, which becomes immunogenic for T-cells. Subsequently, they activate B cells producing ANCA. Also, hydralazine and its metabolites can accumulate within neutrophils and chemically modify MPO as well as turn neutrophil proteins such as elastase, lactoferrin, and nuclear antigens immunogenic. Another hypothesis is that drugs can cause neutrophil apoptosis with the resulting translocation of ANCA antigens to the cell surface and the production of ANCA. Then, ANCA binds to the membrane-bound antigens and provokes crosslinking of PR3, MPO, and Fcy receptors [2]. The literature also reported that the risk of developing hydralazine-induced ANCA-associated vasculitis is higher with prolonged treatment and increased doses of hydralazine in slow acetylators, females, and patients with thyroid diseases [3]. Hydralazine-induced vasculitis can present with lung manifestations (interstitial lung disease, hemorrhage, etc), pauci-immune glomerulonephritis, and hypocomplementemia. Commonly the patients may have symptoms of both hydralazine-induced lupus and hydralazine-induced vasculitis with positive ANA, ANCA, histone, and MPO antibodies [4]. Interestingly, in our case anti-histone antibodies were negative and only C3 was mildly low. It is obvious that the offending medication has to be stopped although this intervention can not immediately reverse the symptoms. All other treatment modalities are to be chosen with consideration of the severity of the disease. Immunosuppressive medications such as steroids, cyclophosphamide, or rituximab may be acceptable options for patients presenting with severe renal and pulmonary involvement [1]. Unfortunately, complications such as infections and end-stage renal failure similar to those of patients with primary ANCA-associated glomerulonephritis treated with standard therapy can also develop [5].

## Conclusions

Further research on hydralazine-induced vasculitis is required. Recommendations regarding treatment should be made as uncontrolled vasculitis can result in severe morbidity and mortality.
